# West Nile Virus and Wildlife Health

**DOI:** 10.3201/eid0907.030277

**Published:** 2003-07

**Authors:** Peter P. Marra, Sean M. Griffing, Robert G. McLean

**Affiliations:** * Smithsonian Environmental Research Center, Edgewater, Maryland, USA; † National Wildlife Research Center, Fort Collins, Colorado, USA

The West Nile Virus and Wildlife Health Workshop, hosted by the Smithsonian Institution, National Audubon Society, U.S. Geological Survey, and U.S. Department of Agriculture, was held February 5–7, 2003, at the Smithsonian Environmental Research Center in Edgewater, Maryland. The event was attended by more than 100 scientists, who heard 29 speakers and participated in strategy discussions during the 2-day meeting. The main focus of the conference was the present and future impact of West Nile virus on wildlife populations. Talks and discussions emphasized how basic research, public health, and land management can contribute to our understanding of the disease’s impact and spread. A primary objective of this meeting was to develop future research priorities from both basic and applied perspectives.

The conference centered around four main themes: 1) host, vector, and pathogen interactions (disease ecology); 2) vertebrate behavior and ecology; 3) vector behavior and ecology; and 4) modeling and spatial statistics. We describe some of the findings from the meeting. For an in-depth summary of this meeting, please visit the conference website for meeting abstracts and a downloadable conference white paper (available from: URL: www.serc.si.edu/migratorybirds/migratorybirds_index.htm).

West Nile virus (WNV) has spread rapidly across North America since its probable introduction to the New York City area in 1999 (D.J. Gubler, Centers for Disease Control and Prevention, Fort Collins, CO). By December 2002, the Canadian provinces of Saskatchewan, Quebec, Ontario, Nova Scotia, and Manitoba reported dead birds that tested positive for WNV. By winter 2002, only four states in the continental United States remained free of confirmed WNV infection; the virus was expected to reach the West Coast later in the year. WNV has also found its way into tropical regions. One case in a person was reported in 2001 from the Cayman Islands. Additionally, resident birds from Jamaica (January 2002) and the Dominican Republic (November 2002) have shown WNV antibodies. Recent reports note that the virus has also reached Mexico’s Yucatan peninsula. Since 1999, WNV has killed thousands of birds and other wildlife, and the impact on regional wildlife populations is unclear.

A primary theme of the meeting was that we still have much to learn about how WNV is dispersed, transmitted, and amplified by competent vectors and still relatively unknown reservoir hosts. At the time of the conference, WNV had been detected in 37 mosquito species, 157 bird species, horses, 16 other mammals, and alligators (D.J. Gubler, Centers for Disease Control and Prevention, Fort Collins, CO). The *Culex* genus, particularly *Culex pipiens* in the northern United States and *C. quinquefasciatus* in the southern United States, appears to be the most important mosquito group for the avian vector amplification cycle. However, opportunistic mosquito species are probably important bridge vectors to humans, horses, and other deadend hosts. While the avian amplification cycle appears to the most dominant, other cycles may also be occurring at the same time (i.e., in mammal and ticks). Reptiles, amphibians, and associated mosquito vectors may also play important roles (M.J. Turell, The United States Army Medical Research Institute for Infectious Diseases, Fort Detrick, MD, and E. Jacobson, University of Florida, Gainesville, FL). Although mosquitoes appear to be the main vector, other ectoparasites such as ticks, louse flies, and fleas should also be examined as potential vectors.

Several scientists reported that transmission of WNV is more complicated than previously thought. The presence of WNV in avian reproductive organs suggests that vertical transmission may be a possibility (T.S. McNamara, Wildlife Conservation Society, Bronx, NY). WNV in the kidneys leads to cloacal excretion, which may lead to cloacal-oral mouth infection. Bird-to-bird transmission has been demonstrated in the laboratory and may be an important infection route among social birds like the American crow (R.G. McLean, U.S. Department of Agriculture, Fort Collins, CO). Evidence suggests that ingesting infected vertebrates and mosquitoes can infect birds.

The impact of WNV on animal populations is another unknown area. Data from individually marked populations of crows in New York State and Oklahoma (K.J. McGowan, Cornell University, Ithaca, NY; A. Clark, State University of New York-Binghamton, Binghamton, NY; and C.L. Caffrey, National Audubon Society, Ivyland, PA) show that these populations are experiencing important declines after the initial WNV outbreak. Analysis of breeding bird surveys and annual winter bird censuses (Christmas bird count) from a wide array of passerine bird species showed local declines in WNV “hotspots” but no declines at the range-wide scale that can be attributed to WNV (J. Sauer, United States Geological Survey, Laurel, MD; P.P. Marra, Smithsonian Environmental Research Center, Edgewater, MD; and W. Hochachka, Cornell University, Ithaca, NY).

Another important issue discussed at this conference was the secondary impact that pest management might have on organisms not pinpointed for WNV, especially in aquatic environments. This issue is especially important in nature reserves (W.K. Reisen, University of California, Davis, CA).

Modelers attending the meeting stressed the importance of standardizing sampling methods, such as the dead bird surveillance programs operated across the nation by many state health departments. These programs must consistently and conscientiously monitor sampling efforts and report the total sample sizes of dead birds collected, including the number of birds that test negative (D.J. Rogers, Oxford, UK). In addition, a better understanding of the real-world persistence of WNV antibodies in live bird surveillance programs would be useful for virus dispersal models.

Scientists at the meeting felt strongly that we need to closely monitor how WNV impacts organisms in tropical regions, including humans and the many endemic avian species already threatened or endangered. Species in Hawaii, many of which are still endangered after malaria’s century-old invasion, should be of special concern. WNV is not the first and will not be the last virus to enter our borders. By developing techniques to survey, monitor, and control WNV in wildlife, we prepare ourselves for the next pathogen species. Our experiences with WNV emphasize the need to strengthen and integrate animal monitoring programs with basic research on population and disease ecology. A conference white paper, several review articles, and a list of research priorities are planned as products of this meeting.

